# The transition in the etiologies of hepatocellular carcinoma-complicated liver cirrhosis in a nationwide survey of Japan

**DOI:** 10.1007/s00535-020-01748-x

**Published:** 2020-11-20

**Authors:** Hirayuki Enomoto, Yoshiyuki Ueno, Yoichi Hiasa, Hiroki Nishikawa, Shuhei Hige, Yasuhiro Takikawa, Makiko Taniai, Toru Ishikawa, Kohichiroh Yasui, Akinobu Takaki, Koichi Takaguchi, Akio Ido, Masayuki Kurosaki, Tatsuya Kanto, Shuhei Nishiguchi, Japan Etiology of Liver Cirrhosis Study Group in the 54th Annual Meeting of JSH

**Affiliations:** 1 Division of Gastroenterology and Hepatology, Department of Internal Medicine, Hyogo College of Medicine, Nishinomiya, Japan; 2 Department of Gastroenterology, Faculty of Medicine, Yamagata University, Yamagata, Japan; 3 Department of Gastroenterology and Metabology, Ehime University Graduate School of Medicine, Toon, Japan; 4 Center for Clinical Research and Education, Hyogo College of Medicine, Nishinomiya, Japan; 5 Department of Hepatology, Sapporo Kosei General Hospital, Sapporo, Japan; 6 Division of Hepatology, Department of Internal Medicine, Iwate Medical University, Morioka, Japan; 7 Internal Medicine, Institute of Gastroenterology, Tokyo Women’s Medical University, Tokyo, Japan; 8 Department of Gastroenterology and Hepatology, Saiseikai Niigata Daini Hospital, Niigata, Japan; 9 Department of Gastroenterology and Hepatology, Kyoto Prefectural University of Medicine, Kyoto, Japan; 10 Department of Gastroenterology and Hepatology, Okayama University Graduate School of Medicine, Dentistry and Pharmaceutical Sciences, Okayama, Japan; 11 Department of Hepatology, Kagawa Prefectural Central Hospital, Takamatsu, Japan; 12 Digestive and Lifestyle Diseases, Kagoshima University Graduate School of Medical and Dental Sciences, Kagoshima, Japan; 13 Department of Gastroenterology and Hepatology, Musashino Red Cross Hospital, Musashino, Japan; 14 Hepatitis Information Center, The Research Center for Hepatitis and Immunology, National Center for Global Health and Medicine, Tokyo, Japan; 15 Department of Gastroenterology, Kano General Hospital, 7-5-15, Tenjin-bashi, Kita-ku, Osaka, 531-0041 Japan

**Keywords:** Hepatocellular carcinoma, Cirrhosis, Etiology, Nationwide survey

## Abstract

**Background:**

We recently reported the real-world changes in the etiologies of liver cirrhosis (LC) based on nationwide survey data and assessed the etiologies of LC with hepatocellular carcinoma (HCC).

**Methods:**

Fifty-five participants from 68 institutions provided data on 23,637 patients with HCC-complicated LC. The changing trends in etiologies were assessed. We further analyzed the data from 29 hospitals that provided the annual number of newly identified HCC-complicated LC patients from 2008 to 2016 (*N* = 9362) without any missing years and assessed the transition in the real number of newly identified HCC-complicated LC cases.

**Results:**

In the overall cohort, hepatitis C virus (HCV) infection (60.3%) and hepatitis B virus (HBV) infection (12.9%) were the leading and third-most common causes of HCC-complicated LC in Japan, respectively. HCV infection was found to be the leading cause throughout Japan. The rate of viral hepatitis-related HCC decreased from 85.3 to 64.4%. Among non-viral etiologies, notable increases were observed in nonalcoholic steatohepatitis (NASH)-related HCC (from 1.5 to 7.2%) and alcoholic liver disease (ALD)-related HCC (from 8.5 to 18.6%). Regarding the real number of newly diagnosed patients, the number of patients with viral hepatitis-related HCC decreased, while the number of patients with non-viral HCC, particularly NASH-related HCC, increased.

**Conclusions:**

Viral hepatitis has remained the main cause of HCC in Japan. However, the decrease in viral hepatitis-related HCC, particularly HCV-related HCC highly contributed to the etiological changes. In addition, the increased incidence of non-viral HCC, particularly NASH-related HCC, was involved in the changing etiologies of HCC-complicated LC in Japan.

**Electronic supplementary material:**

The online version of this article (10.1007/s00535-020-01748-x) contains supplementary material, which is available to authorized users.

## Introduction

Viral hepatitis, which causes liver cirrhosis (LC) and hepatocellular carcinoma (HCC), is a worldwide health concern [1, 2]. Because of recent advances in the treatment of viral hepatitis, the World Health Organization aimed to achieve a 90% reduction of new infections and 65% reduction of viral hepatitis-related mortality by 2030 [3]; however, only a limited number of countries are expected to reach the goal [4]. In Japan, the prevalence of hepatitis B virus (HBV) and hepatitis C virus (HCV) are high, and several measurements for viral hepatitis have been officially conducted by the Ministry of Health, Labour and Welfare (MHLW) [5, 6]. Japan is considered to be one of the most successful countries in the world with regard to the suppression of viral hepatitis [4].

The aim of the antiviral treatments is to improve the prognosis of viral hepatitis-infected patients through precautions to prevent the development of fatal complications, such as liver failure and HCC [7, 8]. In our recent nationwide survey to assess the transition in the etiologies of LC in Japan [9], we found a decrease in hepatitis virus-related LC, particularly in HCV-related LC and an increase in non-viral LC. However, the incidence of HCC development differed among the etiologies, and viral hepatitis-related LC is known to show a higher risk of HCC development in comparison to non-viral LC [10]. Thus, the etiology of HCC is also clinically relevant. In the present study, we used nationwide data to analyze the transition in the etiologies of HCC-complicated LC.

## Patients and methods

### The diagnosis of LC and HCC and classification of the etiologies

As described in our previous report [9], this survey was conducted in the 54th Japan Society of Hepatology (JSH) meeting. The diagnosis and etiological classifications were based on two textbooks edited by the JSH (“Clinical Practice Guidelines for Management of Chronic Hepatitis and Cirrhosis 2016” [11] and “Textbook of Hepatology, 2nd Edition 2016” [12]). These books were published for daily clinical practice and allowed a diagnosis of LC according to clinical findings (physical, laboratory, and imaging results), due to the difficulties associated with performing routine liver biopsy in the clinical setting. The presence of HCC was diagnosed based on imaging modalities such as ultrasound, CT and MRI; a histological evaluation was not mandatory, so the valid period for the diagnosis of LC by a liver biopsy was not specified. The classifications were defined as follows [9, 11, 12]: (1) hepatitis viral infection (HBV, HCV, and HBV + HCV co-infection); (2) alcoholic-related liver disease (ALD); (3) autoimmune hepatitis (AIH); (4) cholestasis; (5) hereditary metabolic diseases; (6) congestive liver diseases; (7) drug-induced liver injury; 8) specific infectious diseases; (9) nonalcoholic steatohepatitis (NASH); and (10) cryptogenic liver cirrhosis. Hepatitis viral infection was diagnosed according to the generally defined criteria (HBV infection; positivity for HBs antigen and HCV infection; positivity for HCV antibodies and HCV RNA). The classifications of non-viral LC were defined as follows: (1) LC due to primary biliary cholangitis (PBC) and primary sclerosing cholangitis were classified as cholestasis-related LC; (2) cases that did not fulfill the criteria for either ALD-related LC (alcoholic intake ≥ 60 g/day) or NASH-related LC (alcoholic intake < 30 g in men and < 20 g in women) were classified as cryptogenic LC; and (3) cases involving overlapping AIH and PBC were classified as AIH-related LC [9].

### The data accumulation of HCC patients

A nationwide survey to investigate the etiologies of LC throughout Japan was conducted in the 54th Annual Meeting of the JSH. A total of 68 presenters showed data from 79 hospitals (see “Appendix” section). In our nationwide survey [9], the LC patients were diagnosed according to the “Clinical Practice Guidelines for Management of Chronic Hepatitis and Cirrhosis 2016” [11], and we analyzed a total of 45,834 LC cases for which the diagnosis year was available. In the survey for the etiologies of LC, the participants also provided the available data regarding the numbers of HCC-complicated LC patients, and 55 participants in 66 institutions with a nationwide geographical distribution provided the transition of etiologies of HCC-complicated LC. The retrospective study with clinical records was approved by the appropriate ethics committees. Each institution rechecked its own clinical records, and the data collection was completed in March 2019 [9].

**Fig. 1:**
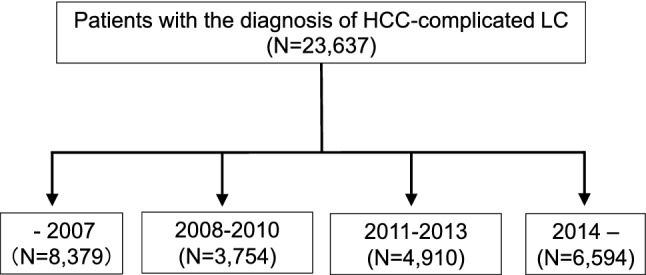
Flowchart of the assessment of the general trends in the etiologies of HCC patients. Data on a total of 23,637 HCC-complicated LC patients were accumulated from a nationwide survey. The patients were classified into four groups according to year of the diagnosis (–2007, 2008–2010, 2011–2013, and 2014–), and the general trends in the etiologies of HCC-complicated LC were assessed

A flowchart to assess the etiological distribution and transition of the HCC-complicated LC was shown in Fig. 1. In line with the previous report [9], we first assessed the etiologies of all HCC patients provided (*N* = 23,637), and then classified the patients into four groups (diagnosed in –2007, 2008–2010, 2011–2013, and 2014–), as the official surveillance of Japan was conducted by the MHLW every three years (2008, 2011, and 2014). Unlike the LC data in our previous report [9], the year in which HCC was diagnosed was provided in all HCC cases. Most of the institutions showed data from after the year when HCV infection had been serologically detectable (from 1992), but the oldest data included cases diagnosed in 1976 by detecting HBV and HCV using preserved sera.

**Fig. 2:**
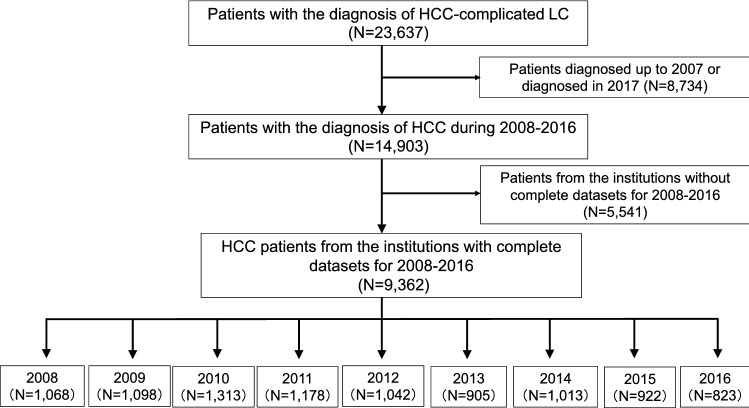
Flowchart of the assessment of the annual numbers of newly diagnosed HCC patients. In this study, we aimed to estimate the transition in the real number of cases of newly identified HCC-complicated LC. Most of the hospitals supplied data up to 2016, since the deadline for the abstract submission was set in December 2017. Among the contributing hospitals, 29 had comprehensive data on the number of newly identified LC patients for 2008–2016 (*N* = 9362). We used these data to estimate the trends in the annual number of newly diagnosed HCC cases in designated hospitals in 2008–2016

Among the participating institutions, 29 were able to provide the consecutive annual data on the number of newly identified HCC-complicated LC patients from 2008 to 2016. In accordance with the survey of the LC patients, we used the data of these 29 hospitals to estimate the alterations in the real numbers of patients with newly developed HCC in the same institutions from 2008 to 2016 (Fig. 2).

### Statistical analyses

The frequency among multiple groups was compared by the Chi-square test. Regarding the frequencies of etiologies among the eight areas (Hokkaido, Tohoku, Kanto, Chubu, Kinki, Chugoku, Shikoku and Kyushu areas), the areas with significantly higher or lower rates were subsequently determined by a residual analysis [9].

## Results

### Overall results regarding the etiologies of HCC and geographic differences

**Fig. 3:**
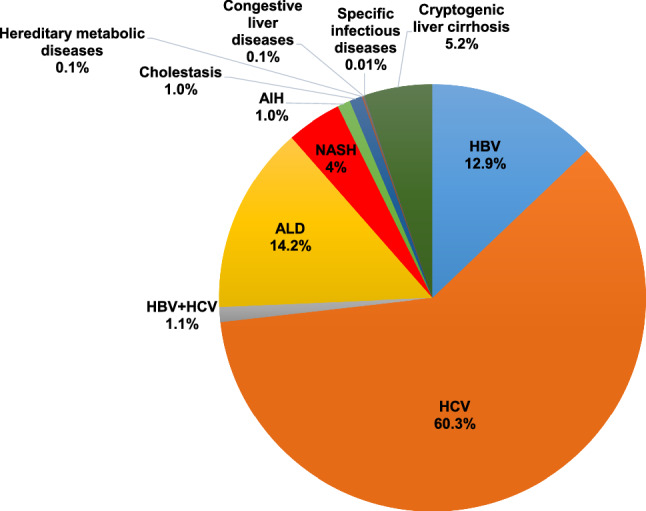
Overall results regarding the etiologies of LC in the collected data. The 23,637 patients included 15,803 (66.8%) male patients and 7834 (33.2%) were female. HCV mono-infection (60.3%) and HBV mono-infection (12.9%) were the leading cause and the third-most common cause of HCC in Japan, respectively. Alcoholic-related liver disease (14.2%) and NASH (4.2%) were also important causes of HCC in Japan; however, viral hepatitis, particularly HCV, remained the dominant cause in the current survey

**Fig. 4:**
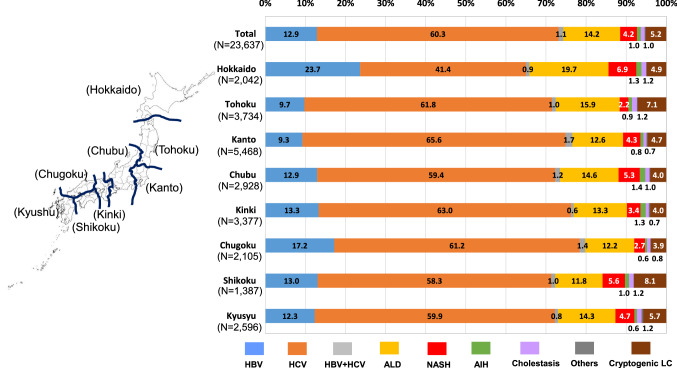
Geographic differences in the etiologies of HCC-complicated LC. The etiologies of HCC-complicated LC in the different geographic areas are shown. HCV infection was the leading cause of HCC-complicated LC in all areas. The rate of the ‘others’ etiology was quite low in all areas (< 0.5%) and was not described in the figure

A total of 23,637 cases [male, *n* = 15,803 (66.8%); female male, *n* = 7834 (33.2%)] were accumulated and used to assess the etiologies of HCC-complicated LC in Japan (Fig. 3). HCV infection (60.3%) and HBV infection (12.9%) were the leading cause and the third-most common cause of HCC in Japan, respectively. Although ALD (14.2%) and NASH (4.2%) were found to be important causes of HCC, viral hepatitis-related HCC was prominent in Japan. In line with the methods of the previous study, we divided Japan into eight areas (Fig. 4, left panel), and HCV infection was found to be the leading cause of HCC in all areas of Japan. The summarized results regarding the differences among the areas were as follows (Fig. 4, right panel). The Hokkaido and Chugoku areas showed high rates of HBV-related HCC (*p* < 1.0 × 10^–15^ and *p* < 1.0 × 10^–9^, respectively), while the Tohoku and Kanto areas showed low rates (*p* < 1.0 × 10^–9^ and *p* < 1.0 × 10^–15^, respectively). The Kanto and Kinki areas showed high rates of HCV-related HCC (*p* < 1.0 × 10^–9^ and *p* < 1.0 × 10^–5^, respectively), while the Hokkaido area showed a low rate (*p* < 1.0 × 10^–15^). The Hokkaido and Tohoku areas showed high rates of ALD-related HCC (*p* < 1.0 × 10^–12^ and *p* < 1.0 × 10^–2^, respectively), while the Kanto, Chugoku, and Shikoku areas showed low rates (*p* < 1.0 × 10^–3^, *p* < 1.0 × 10^–2^ and *p* < 1.0 × 10^–2^, respectively). The Hokkaido, Chubu and Shikoku areas showed high rates of NASH-related HCC (*p* < 1.0 × 10^–10^, *p* < 1.0 × 10^–2^ and *p* < 1.0 × 10^–2^, respectively), while the Tohoku and Chugoku areas showed low rates (*p* < 1.0 × 10^–10^ and *p* < 1.0 × 10^–2^, respectively).

Since the current study focused on the etiological transition, the data on patients’ ages were not obtained from some institutions. However, the available data of 16,092 patients suggested an increase in the HCC patient age over the past decade (Supplementary Fig. 2a). Increasing trends in the age of HCC patients during the recent decade were also suggested in different geographic areas of Japan (Supplementary Fig 2b–i).

### The decreased rate of viral hepatitis-related HCC

**Fig. 5:**
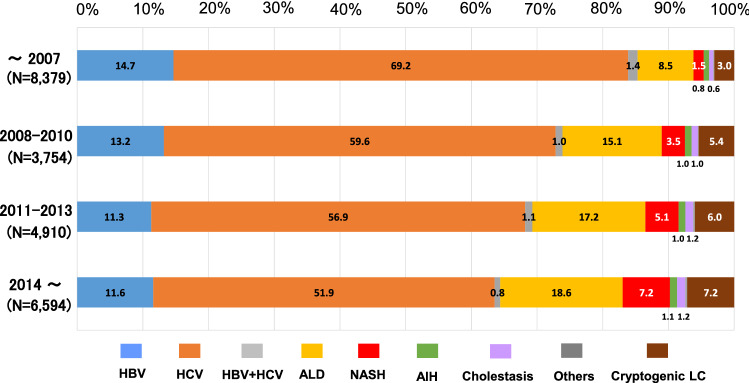
Transition of the distribution regarding the etiologies of HCC-complicated LC. The rate of viral hepatitis-related HCC, particularly HCV-related HCC, showed a remarkable decrease during the last decade, while the rate of non-viral LC increased. However, the rate of viral hepatitis-related HCC remained > 60% even in the data after 2014. The rate of the ‘others’ etiology was quite low in all periods (< 0.5%) and was not described in the figure

We further assessed the transition in the etiologies of HCC. In the data before 2008, the rate of viral hepatitis-related HCC was quite high (85.3%). The rate of viral hepatitis-related LC tended to decrease over time (73.9% in the 2008–2010 data and 69.3% in the 2011–2013 data). In the data from 2014 and thereafter, the rate of viral hepatitis-related HCC further decreased but still remained high (64.4%) (Fig. 5). When the oldest data (before 2008) and the most recent data (2014 and thereafter) were compared, the rates of HBV (14.7%), HCV (69.2%) and HBV + HCV (1.4%) dropped to 11.6% (*p* < 1.0 × 10^–10^), 51.9% (*p* < 1.0 × 10^–10^) and 0.8% (*p* < 0.05), respectively. Among the etiologies of non-viral HCC, the rate of NASH-related HCC showed an approximately fivefold increase from 1.5 to 7.2% (*p* < 1.0 × 10^–10^). The rate of ALD-related HCC also increased from 8.5 to 18.6% (*p* < 1.0 × 10^–10^).

**Fig. 6:**
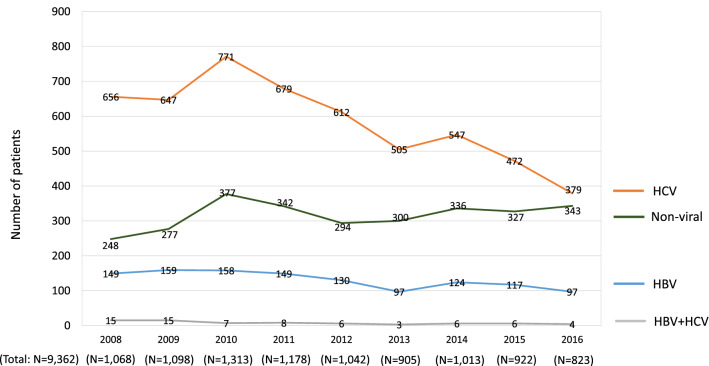
The transition in the number of cases of newly diagnosed HCC-complicated LC. A total of 9362 cases of newly diagnosed HCC in the 29 designated hospitals were analyzed according to the year of diagnosis. In 2008–2016, the real numbers of patients with viral hepatitis-related HCC, particularly HCV-related HCC, decreased, whereas the real number of patients with non-viral LC gradually increased

To assess the transition in HCC etiologies in detail, we also evaluated the changes in the real numbers of patients. We analyzed the data of the 29 hospitals with records that included the annual number of newly identified HCC patients each year, without any missing years, from 2008 to 2016 (see Fig. 2). A total of 9362 cases with 6320 male patients (67.5%) and 3042 female patients (32.5%) were included. A comparison between the data in 2008 and 2016 revealed that the numbers of newly diagnosed HCC patients with HBV mono-infection decreased from 149 to 97 (Fig. 6; blue line). The numbers of HCC patients with HCV mono-infection also decreased from 656 to 379 (Fig. 6; orange line). In contrast, the numbers of patients who were newly diagnosed with non-viral HCC increased from 248 to 343 (Fig. 6; green line).

### The transition of etiologies in non-viral HCC patients

**Fig. 7:**
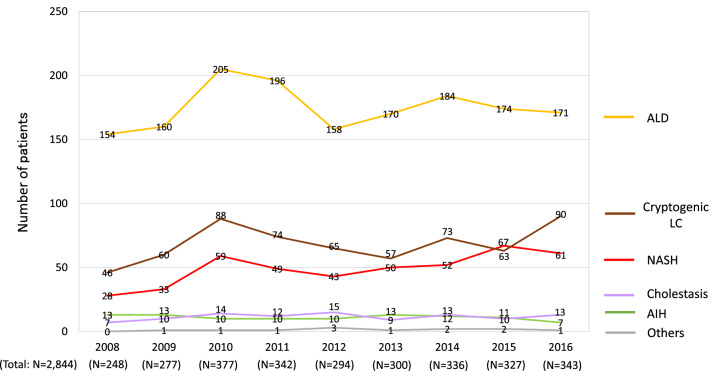
The transition in the number of patients who were diagnosed with HCC of non-viral etiologies. The number of cases of newly diagnosed non-viral HCC increased in real numbers from 2008 to 2016 (Fig. 6), and more detailed data are shown. The numbers of cases of ALD-related HCC, NASH-related HCC and cryptogenic LC were suggested to increase from 2008 to 2016, and an increase in NASH-related HCC was particularly evident

As the real number of non-viral HCC cases was suggested to increase in the recent decade (Fig. 6), we further assessed the transition in newly identified non-viral HCC cases in greater detail (Fig. 7). The number of ALD-related HCC cases showed a mild increase from 154 to 172 (Fig. 7; gold line). The newly diagnosed patients with NASH-related HCC showed a more than twofold increase from 28 to 61 (Fig. 7; red line). The number of patients diagnosed with cryptogenic LC increased from 46 to 90 (Fig. 7; brown line). These findings suggested that, in Japan, newly diagnosed non-viral HCC, particularly NASH-related HCC, increased in real numbers from 2008 to 2016.

## Discussion

HCC is a major complication of LC patients. In the present study, we showed the changing etiology of HCC-complicated LC in Japan. Our previous report on the survey on the etiologies of LC revealed that the contribution of viral hepatitis remarkably decreased, and the ratio of HCV-related LC dropped to < 50% after 2014. We considered that this was due to recent advances in treatment for viral hepatitis [13–18] and official measures that are provided in Japan such as the financial support system for patients to receive antiviral treatment [5, 6]. However, in the current study, viral hepatitis was shown to remain a major cause of HCC in Japan, although the contribution had gradually decreased during the recent decade. Viral hepatitis-related LC is known to be associated with a high rate of HCC development in comparison to non-viral LC; and our results are consistent with this fact, and showed that viral hepatitis remains an important etiology of HCC in Japan.

The etiologies of HCC in Japan have been the subject of previous studies [19–22]. In the surveillance of LC etiology in 2008 [19], the etiological distribution of HCC-complicated LC patients was reported as follows: HBV (14.1%), HCV (73.1%), ALD (6.3%), and NASH (1.6%). Although the rate of HBV and HCV co-infection was not mentioned, approximately 90% of the patients with HCC were diagnosed with hepatitis virus infection. In the results of a nationwide survey on HCC in 2009 [20], the distribution of the major etiologies was reported to be as follows: HBV (14.1%), HCV (66.3%), HBV + HCV (3.7%), ALD (7.2%), and NAFLD (2.0%). In our study, the etiologies of HCC in patients diagnosed before 2008 were as follows: HBV (14.7%), HCV (69.2%), HBV + HCV (1.4%), ALD (8.5%), and NASH (1.5%) (Fig. 5). A recent report also provided similar results regarding the data of HCC patients diagnosed in 2008 or 2009 [21]. The comparable findings of the different surveys, including the current study, are suggested to appropriately reflect the real-world data of Japanese HCC patients around 10 years previously. In the current study, we found an increase in non-viral HCC (Fig. 5), and ALD-related HCC and cryptogenic HCC were calculated to be responsible for approximately 55 and 20% of non-viral HCC, respectively (Supplementary Fig. 1). These results were—to some extent—different from the results of the recent nationwide survey on non-viral HCC [22], which showed a lower rate of ALD-related HCC (32.3%) and a higher rate of HCC with unclassified and other etiologies (46.7%). The discrepancies may have been caused by the different definitions for the etiological classifications in each study, as the rate of non-viral HCC among all HCC cases was comparable in the two studies, at 32.5% in the previous study [22] and 35.6% in the current study (see the HCC patients diagnosed in 2014 and thereafter in Fig. 5). Our results were also consistent with various studies that assessed the transition in the etiologies of HCC in Japan [23, 24]. In addition, we showed that HCV infection still maintained an important role in the clinical practice of HCC, despite an increase in non-viral HCC, and the results seemed to be consistent with the data reported from outside Japan [25–28]. These findings suggested that our results appropriately reflect recent trends in the etiologies of HCC.

The current study included some unique results. This study is the most recent nationwide survey that analyzed a large number of patients with both viral HCC and non-viral HCC. In addition, we analyzed the differences in the etiologies of HCC among various areas, which have not been routinely assessed. Although HCV-related HCC was the leading cause in all areas (Fig. 4), each area had its own characteristics, which was similarly observed in previous studies on the etiologies of LC [9]. Furthermore, similarly to our previous study [9], we analyzed the data from designated hospitals that were able to provide the annual numbers of newly identified HCC patients from 2008 to 2016. The transition in the new HCC patients in the same hospitals suggested that the decrease in viral hepatitis-related HCC and the increase in non-viral HCC reflected both changes in the rates of each etiology (Fig. 5) and in the real numbers of newly identified HCC patients (Fig. 6). However, we should note that the absolute number of non-viral HCC patients in Japan was smaller than the number of viral hepatitis-related HCC patients. The number of newly diagnosed cases of non-viral HCC, particularly NASH-related HCC, increased in real numbers (Fig. 7); however, the changes were relatively mild in comparison to the remarkable decrease in HCV-related HCC (Fig. 6). In light of the data with real numbers, the decrease in viral hepatitis-related HCC, particularly HCV-related HCC, was suggested to contribute more strongly to the changing distribution of the HCC etiologies in Japan in comparison to the increase in non-viral HCC cases.

As described in the previous paper [9], because of the high prevalence of viral hepatitis, various measures against viral hepatitis have been implemented at the national level, and Japan is considered to be one of the most successful countries in the world with regard to the suppression of viral hepatitis. HCV-infected patients in Japan tend to be elderly individuals with long-term infection, since HCV infection is thought to have been widespread approximately 60 years ago [29]. Although our current data were only obtained from Japan, the transition in the etiologies, with a reduction in the numbers of viral hepatitis-related HCC cases, particularly HCV-related HCC, could help predict the future changes in the etiologies of HCC in other countries [30].

The present study was associated with some limitations. First, we only surveyed the numbers of patients with HCC-complicated LC. Our nationwide data demonstrated a difference in the etiology between the whole LC patients and the HCC-complicated LC patients, and viral hepatitis remains an important etiology of HCC in Japan. Previous reports that focused on specific patients, such as non-viral HCC cases, have provided significant findings for physicians [20, 22]. We, therefore, feel that the current results focusing on LC patients could be clinically useful. However, analyzing the whole HCC patient cohort, including non-cirrhotic patients, would be quite informative. Second, although the current study was the latest nationwide survey, we did not include the detailed clinical data. Thus, we were unable to include data that might be affected by the etiologies, including the composition of HCC by cause and sex ratio of HCC by cause. In addition, the number of patients was not directly associated with the prevalence of liver diseases, as our survey did not include the data of the general population. Third, LC and HCC were determined based on the clinical diagnosis, without histological assessments. Finally, we analyzed the hospitals with annual data on the number of newly diagnosed HCC patients for every year from 2008 to 2016 (Figs. 6, 7). In these hospitals, the real numbers of new HCC patients were considered to have been regularly recorded, independently of the current survey, and may simulate prospectively accumulated data. However, the analysis of a prospectively enrolled cohort would be warranted to precisely assess the transition in the real numbers of HCC patients in Japan.

In summary, viral hepatitis infection was considered to remain a major cause of HCC in Japan; however, its contribution as a cause of HCC has been decreasing during the recent decade. The decrease in viral hepatitis-related HCC, particularly HCV-related HCC, was suggested to highly contribute to the change in the distribution of the etiologies of HCC in Japan.

### Electronic supplementary material

Below is the link to the electronic supplementary material.

Supplementary file1 (PDF 111 KB)

## Appendix

We appreciate the following institutions that participated in the poster symposium of the 54th Annual Meeting of the JSH. Akita University Graduate School of Medicine; Anjo Kosei Hospital; Asahikawa City Hospital; Asahikawa Medical University; Chiba University, Graduate school of medicine (Gastroenterology); Dokkyo Medical University Saitama Medical Center; Ehime University Graduate School of Medicine (Department of Gastroenterology and Metabology); Fukushima Medical University School of Medicine (Department of Gastroenterology); Gunma University Hospital; Hakodate Municipal Hospital; Hiroshima Red Cross and Atomic Bomb Survivors Hospital; Hiroshima University Hospital (Gastroenterology and Metabolism); Hokkaido P.W.F.A.C. Asahikawa-Kosei General Hospital; Hokkaido University Hospital (Department of Gastroenterology and Hepatology); Hyogo College of Medicine (Division of Hepatobiliary and Pancreatic Diseases, Department of Internal Medicine); Hyogo Prefectural Nishinomiya Hospital; Ikeda Municipal Hospital (Department of Gastroenterology); Isesaki Municipal Hospital; Iwate Medical University; Japanese Red Cross Asahikawa Hospital; Japanese Red Cross Date Hospital (Department of Gastroenterology); Jichi Medical University (Department of Medicine, Division of Gastroenterology and Hepatology); Juntendo University Shizuoka Hospital (Gastroenterology and Hepatology); Kagawa Prefectural Central Hospital (Department of Hepatology); Kagawa University (Department of Gastroenterology and Neurology); Kagoshima University Graduate School of Medical and Dental Sciences (Digestive and Lifestyle Diseases, Department of Human and Environmental Sciences); Kanazawa University (Department of Internal medicine/Hepatology and Gastroenterology); Kansai Rosai Hospital; Kawasaki Municipal Tama Hospital; Keio University Hospital; Kiryu Kosei General Hospital; Kumamoto University, Graduate School of Medical Sciences (Department of Gastroenterology and Hepatology); Kurume University School of Medicine (Division of Gastroenterology, Department of Medicine); Kushiro Rosai Hospital; Kusunoki-Hospital; Kyoto Prefectural University of Medicine Graduate School; Maebashi Red Cross Hospital; Mie University Graduate School of Medicine; Mie-chuo Medical Center; Musashino Red Cross Hospital; Nagoya City University Graduate School of Medical Sciences (Department of Gastroenterology and Metabolism); Nara Medical University (Department of 3rd Internal Medicine); National Hospital Organization Kyoto Medical Center (Department of Gastroenterology); Nayoro City General Hospital; Nihon University school of Medicine (Division of Gastroenterology and Hepatology, Department of Internal Medicine); Niigata University, Graduate School of Medical and Dental Sciences (Division of Gastroenterology and Hepatology); Nippon Life Hospital; Ogaki Municipal Hospital; Oita University, Faculty of Medicine (Department of Gastroenterology); Okayama City Hospital (Department of Gastroenterology); Okayama University Graduate School of Medicine, Dentistry and Pharmaceutical Sciences (Department of Gastroenterology and Hepatology); Ome Municipal General Hospital; Osaka City University, Graduate School of Medicine (Department of Hepatology, Department of Endowed Department of Liver Cirrhosis Therapeutics and Department of Premier Preventive Medicine); Osaka Police Hospital; Public Tomioka General Hospital; SAGA university (Division of Metabolism and Endocrinology Depertment of Internal Medicine); Saga-Ken Medical Centre Koseikan; Saiseikai Niigata Daini Hospital; Saitama Medical Center; Saitama Medical University; Sapporo Kosei General Hospital; Shibukawa Medical Center; Shiga University of Medical Science (Department of internal medicine, gastroenterology),; Shimane University Hospital; Shinshu University School of Medicine; Takasaki General Medical Center; Teine Keijinkai Hospital; Tohoku University Hospital; Tokyo Kyosai Hospital; Tokyo Medical University Ibaraki Medical Center; Tokyo Women's Medical University (Department of Internal Medicine and Gastroenterology); Tomakomai City Hospital; Toranomon Hospital; Tottori University Hospital; University of Fukui, School of Medical Sciences (Second Department of Internal Medicine); University of Ryukyus; University of Yamanashi, Faculty of Medicine (First Department of Internal Medicine); Yamagata University, Faculty of Medicine (Department of Gastroenterology); Yamaguchi University Graduate School of Medicine (Department of Gastroenterology and Hepatology).

## Abbreviations

LC: Liver cirrhosis
HCC: Hepatocellular carcinoma
HBV: Hepatitis B virus
HCV: Hepatitis C virus
MHLW: Ministry of Health, Labour and Welfare
JSH: Japanese Society of Hepatology
ALD: Alcoholic-related liver disease
AIH: Autoimmune hepatitis
NASH: Nonalcoholic steatohepatitis
PBC: Primary biliary cholangitis
