# Supplementary material for: The transition in the etiologies of hepatocellular carcinoma-complicated liver cirrhosis in a nationwide survey of Japan
Source: J Gastroenterol. 2020 Nov 20;56(2):158–67. doi: 10.1007/s00535-020-01748-x (PMC7862502; doi:10.1007/s00535-020-01748-x)
Supplement: Supplementary file 1 — Supplementary file1 (PDF 111 KB) [file 535_2020_1748_MOESM1_ESM.pdf]

Supplementary Material

Enomoto H, et al.; The transition in the etiologies of hepatocellular carcinoma-complicated liver cirrhosis in a nationwide survey of Japan

Supplementary Figure 1: Transition in the etiologies of non-viral hepatocellular carcinoma

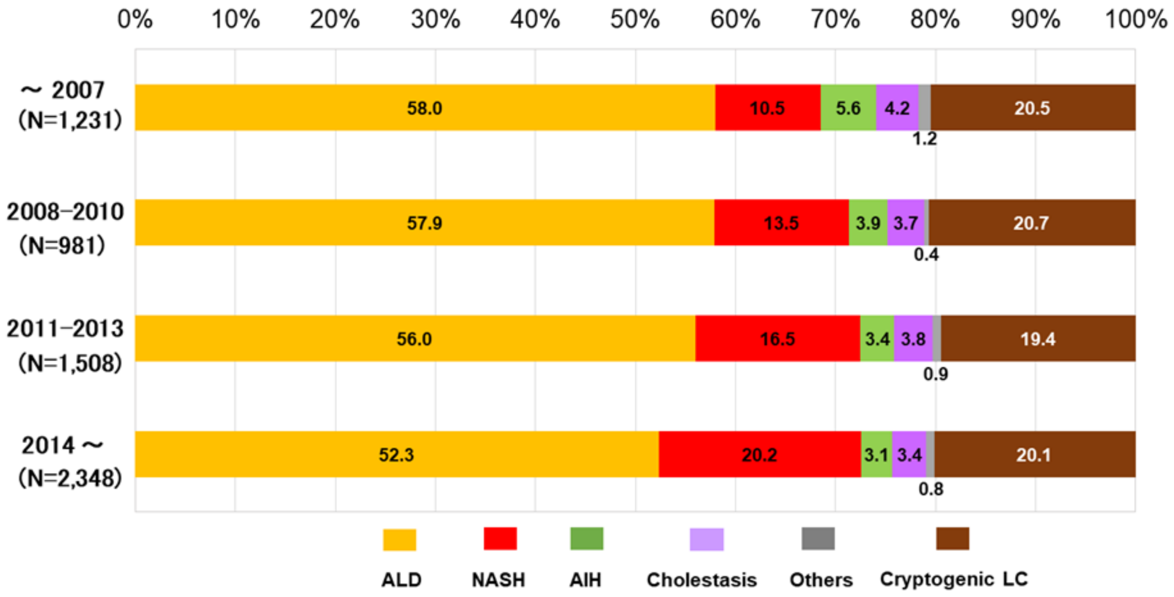

The transition in the etiologies of non-viral hepatocellular carcinoma (HCC) are graphically shown. In the current study, ALD-related HCC and cryptogenic HCC were calculated to be responsible for approximately 55% and 20% of non-viral HCC, respectively.

ALD: alcoholic liver disease; NASH: nonalcoholic steatohepatitis; AIH: autoimmune hepatitis

Supplementary Figure 2: Transition in the age of hepatocellular carcinoma-complicated liver cirrhosis patients

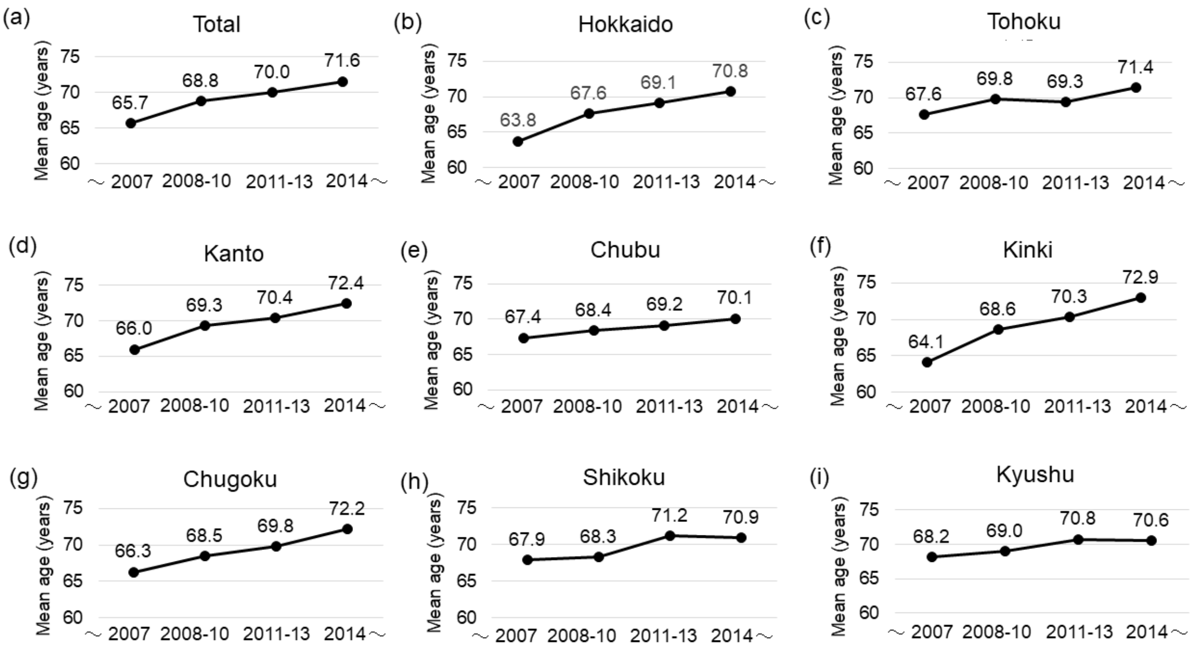

(a) The available data of 16,092 patients with hepatocellular carcinoma (HCC)-complicated liver cirrhosis (LC) suggested an increase in the patient age over the past decade. (b-i) Increasing trends in the age of HCC patients during the recent decade were also suggested in different geographic areas of Japan, including (b) Hokkaido (N=1,985), (C) Tohoku (N=1,270), (d) Kanto (N=2,527), (e) Chubu (N=1,472), (f) Kinki (N=2,984), (g) Chugoku (N=2,105), (h) Shikoku (N=1,387) and (i) Kyushu (N=2,362).
